# Supplementary material for: Seasonal modulation of phytoplankton biomass in the Southern Ocean
Source: Nat Commun. 2020 Oct 23;11:5364. doi: 10.1038/s41467-020-19157-2 (PMC7584623; doi:10.1038/s41467-020-19157-2)
Supplement: Supplementary file 1 — Supplementary Information [file 41467_2020_19157_MOESM1_ESM.pdf]

Supplementary information for

Seasonal modulation of phytoplankton biomass in the Southern Ocean

Lionel A. Arteaga[1,2*], Emmanuel Boss [3], Michael J. Behrenfeld [4], Toby K. Westberry [4], Jorge L. Sarmiento [1]

(*) Corresponding author

Afiliations:

- (1) Program in Atmospheric and Oceanic Sciences, Princeton University, 300 Forrestal Rd, Princeton, NJ, USA,
- (2) NASA Global Modeling and Assimilation Office, Universities Space Research Association, Columbia, MD, USA.
- (3) School of Marine Sciences, 5706 Aubert Hall, University of Maine, Orono, Maine 04469−5741, USA.
- (4) Department of Botany and Plant Pathology, Cordley Hall 2082, Oregon State University, Corvallis, Oregon 97331−2902, USA.

For the present study, BGC-Argo float data deployed by the SOCCOM program between 2012 and 2019 was analyzed altogether and subdivided into environmental zone as explained in the Methods section. The Southern Ocean presents a clear spatial gradient in surface mixed layer biogeochemical properties (temperature, oxygen, and nitrate) across the four environmental zones defined in this study (Figure S1). Float-based phytoplankton biomass and growth variables for the mixed layer are initially obtained for each individual float profiles (Figure S2 and S4), and subsequently averaged to represent the integrated signal of the Southern Ocean and subregions (STZ, SAZ, PAZ, and SIZ) (Figure 1 and 2). The uncertainty in the seasonality of Southern Ocean division rate and phytoplankton net accumulation rate

is computed as the standard deviation of the multi-annual time series of μ and r (Figure 3). Annual climatologies of float-sampled mean mixed layer nitrate, up-to-date compiled dissolved iron observations[1] (Figure S5), and satellite based mixed layer light estimates (Figure S6) were produced and analyzed for each environmental zone in conjunction with temporal changes in phytoplankton biomass.

Individual float-based estimates of phytoplankton division rates (μ) and net accumulation rates based on changes in mixed layer biomass concentration (r_{mld}) and integrated inventory (r_{int}) were obtained as detailed in the Methods section (Figure S7). The seasonality of r_{mld} is similar to that of r_{int}. However, clear differences exist during periods of mixed layer shoaling or deepening. Net accumulation rates based on the mixed layer integrated inventory of biomass (r_{int}) are higher than rates based on changes in the biomass concentration (r_{mld}) during periods of mixed layer deepening, and vice versa (Figure S8). This pattern is consistent with the expected effect of dilution of the mixed layer phytoplankton concentration during increased surface vertical mixing on the computation of accumulation rates based on biomass concentration, and the expected effect from changes in the vertically integrated water layer on the computation of biomass accumulation based on the integrated phytoplankton carbon inventory in the seasonally varying mixed layer[2,3]. The smoothed time series of r_{int} - r_{mld} and the temporal derivative of the mixed layer (dMLD/dt) are computed as described in the Methods section.

Assessment of division rates estimated by the CbPM

Division rate (μ) outputs from the CbPM were compared against a productivity algorithm parameterized specifically for Southern Ocean waters (Arrigo2008)[4]. The Arrigo2008 algo-

rithm computes division rates as:

$$\mu(z,t) = G_{\max}(t) \times L(z,t). \tag{1}$$

$$G_{\max}(t) = G_0 \; e^{rT(t)} \tag{2}$$

where, following the Arrigo2008 (Equation 8 and 9) notation[4], μ at a given time (t) and depth (z) depends on $G_{\max}(t)$, the temperature (T) dependent upper limit to net phytoplankton growth rate (i.e., division rate) and an irradiance limitation term (L). G_0 is the phytoplankton net growth rate at 0°C (0.59 d^{-1}) and r is a rate constant (0.0633 °C^{-1}) that determines the sensitivity of $G_{\max}(t)$ to temperature. The light limitation term, $L(z,t)$, is calculated for each depth and each time step as:

$$L(z,t) = 1 - e^{-\frac{\mathrm{PUR}(z,t)}{E'_k(z,t)}} \tag{3}$$

where PUR is the photosynthetically usable radiation (assumed equivalent to PAR) and E'_k is the spectral photoacclimation parameter (see Equations 10–14 of the Arrigo2008 algorithm description for details on the computation of these parameters)[4].

The temporal evolution of division rate estimated in our study by the CbPM agrees well with that estimated from the Southern Ocean-aimed formulation of Arrigo2008 (Figure S9a). In particular, the 'timing' (increase/decrease) of both estimates of μ follows a similar seasonal cycle over the interannual time series of profiling floats observations, providing confidence in the in situ-based observation of a temporal lag between division rates and the net biomass rate of change (r) (Figure 1).

The CbPM allows for a decomposition of the nutrient and light controlling effects on μ. Nutrient limitation (low nutrient index) is diagnosed to occur during summer months, in opposite fashion to the annual cycle of the light index (Figure S9b). The impact of nutrient

stress on μ is relatively low (i.e., the nutrient index only decreases to about 0.6) which might be due to not explicitly accounting for iron limitation in the model. However, the timing of low nutrient index in summer and high in winter is consistent with the seasonal expectation of micronutrient availability in the Southern Ocean[5] and therefore provides confidence in the ability of the model to detect the correct seasonality of nutrient limitation in this region. As observed above, a productivity algorithm parameterized specifically for Southern Ocean waters (Arrigo2008)[4] presents a very similar seasonality in μ.

We compare float-based estimates of division rates obtained from the CbPM and Arrigo2008 with division rates estimated from a data base of in situ carbon-14 (^{14}C) net primary productivity measurements[6]. In order to infer division rates from in situ-based measurements of vertically integrated NPP, we computed $\mu = \frac{\mathrm{NPP_{int}}}{\mathrm{C_{phyto}} \cdot \mathrm{Z_{eu}}}$ where $\mathrm{NPP_{int}}$ (mg C m^2 d^{-1}) is vertically integrated ^{14}C-based net primary production over the euphotic depth, $\mathrm{C_{phyto}}$ (mg C m^3) is the mean phytoplankton concentration in the euphotic depth, and Z_{eu} (m) is the euphotic depth. $\mathrm{C_{phyto}}$ and Z_{eu} where obtained from a monthly-resolved climatological merged product of MODIS and CALIOP satellite observations used to infer NPP and marine carbon export[7]. In situ-based estimates of μ are obtained by matching the monthly-resolved satellite-based climatologies with the same month at wich ^{14}C NPP measurements were obtained. The in situ-based estimates of division rate show a coherent global pattern, with high μ in the equatorial Pacific Ocean and at high latitudes, and low μ in subtropical regions (Figure S10c). Due to reduced in situ observations in the Southern Ocean, we subsample float-based estimates of μ by averaging all float-based estimates within a horizontal radius of 500 km around each in situ observation at the same month of the year. This results in a total of 187 comparable data points south of 30°S.

A strong correlation between float-based and in situ-based estimates of division rates was not initially expected, since ^{14}C NPP measurements were combined with satellite biomass data to infer μ, float and in situ data are not spatially coincident, and both data sets

were monthly matched but do not coincide perfectly in time (they represent different years). Despite these sources of discrepancy between data sets, we find that float-based division rates estimated from the CbPM compare well and more favorably with in situ-based μ ($R^2 = 0.25$, root-mean-square error of the scatter around the least-squares linear fit ($RMSE_{fit}$) = 0.24 d^{-1}, root-mean-square error between linear fit and one-to-one line ($RMSE_{model}$) = 0.35 d^{-1}) (Figure S10a) than estimates from Arrigo2008 ($R^2 = 0.13$, $RMSE_{fit} = 0.19$ d^{-1}, $RMSE_{model}$ = 0.58 d^{-1}) (Figure S10b). These results indicate that the CbPM is able to reasonably predict phytoplankton division rates (μ) in the Southern Ocean, thereby, providing a robust validation constraint for the results presented in this study.

Sensitivity of phytoplankton seasonal bloom in the Southern Ocean

The analysis of the sensitivity of the Southern Ocean phytoplankton seasonal bloom magnitude and net primary production is based on the reconstruction of the climatological phytoplankton loss rate for the Southern Ocean based on a 2-days temporal lag in μ[8] (Figure S11). The reconstruction of the mean loss rate (l) in the Southern Ocean permitted the assessment of the sensitivity of vertically integrated net primary productivity to induced changes in the climatological seasonal cycle of phytoplankton division rate (μ) (Figure S12).

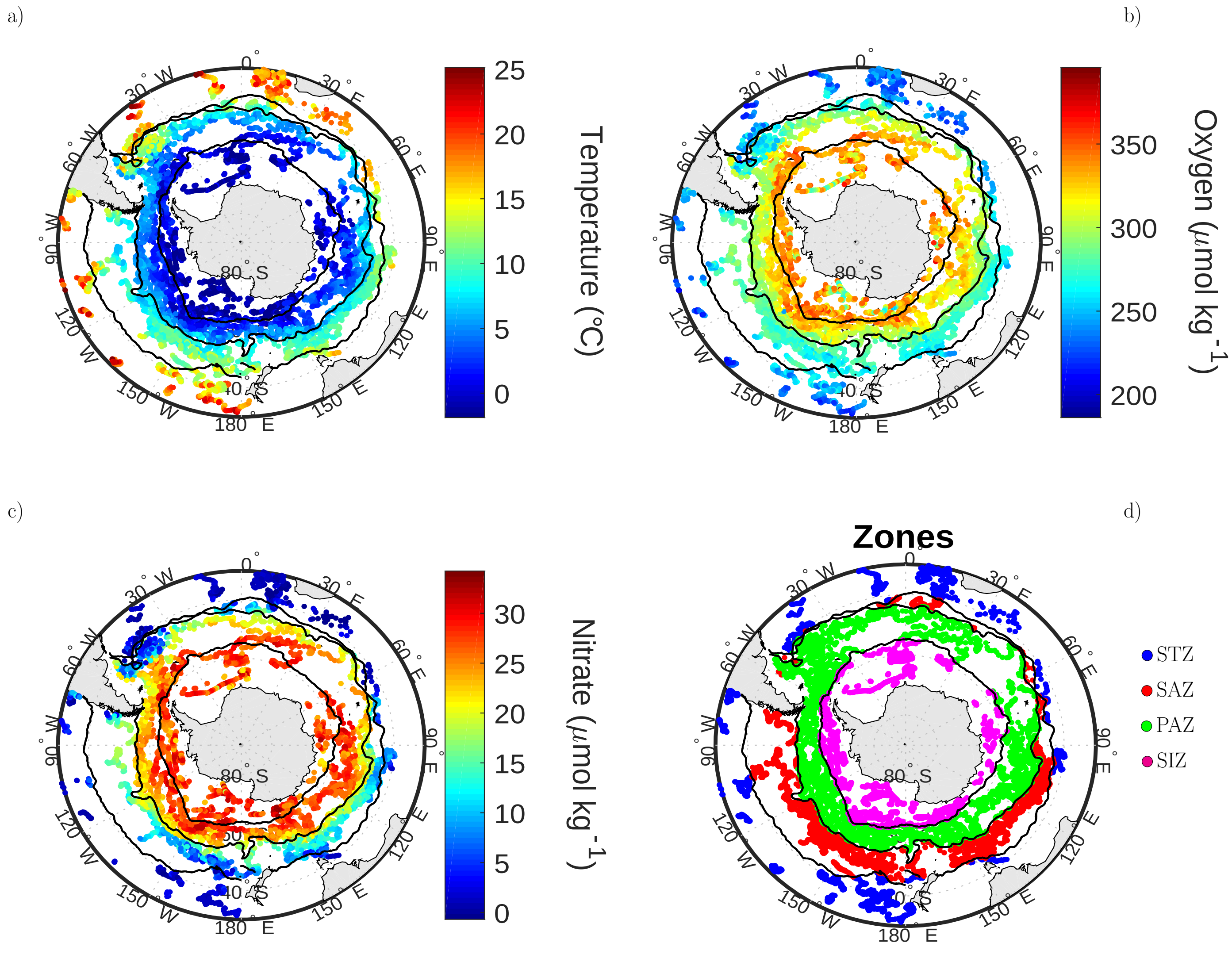

Figure S1: Gradients in mean surface mixed layer (a) temperature, (b) dissolved oxygen, and (c) nitrate concentration measured by the biogeochemical floats. (d) Location of the Southern Ocean environmental zones defined in this study: Subtropical Zone (STZ), Subantarctic Zone (SAZ), Polar Antarctic Zone (PAZ), and Seasonal Ice Zone (SIZ).

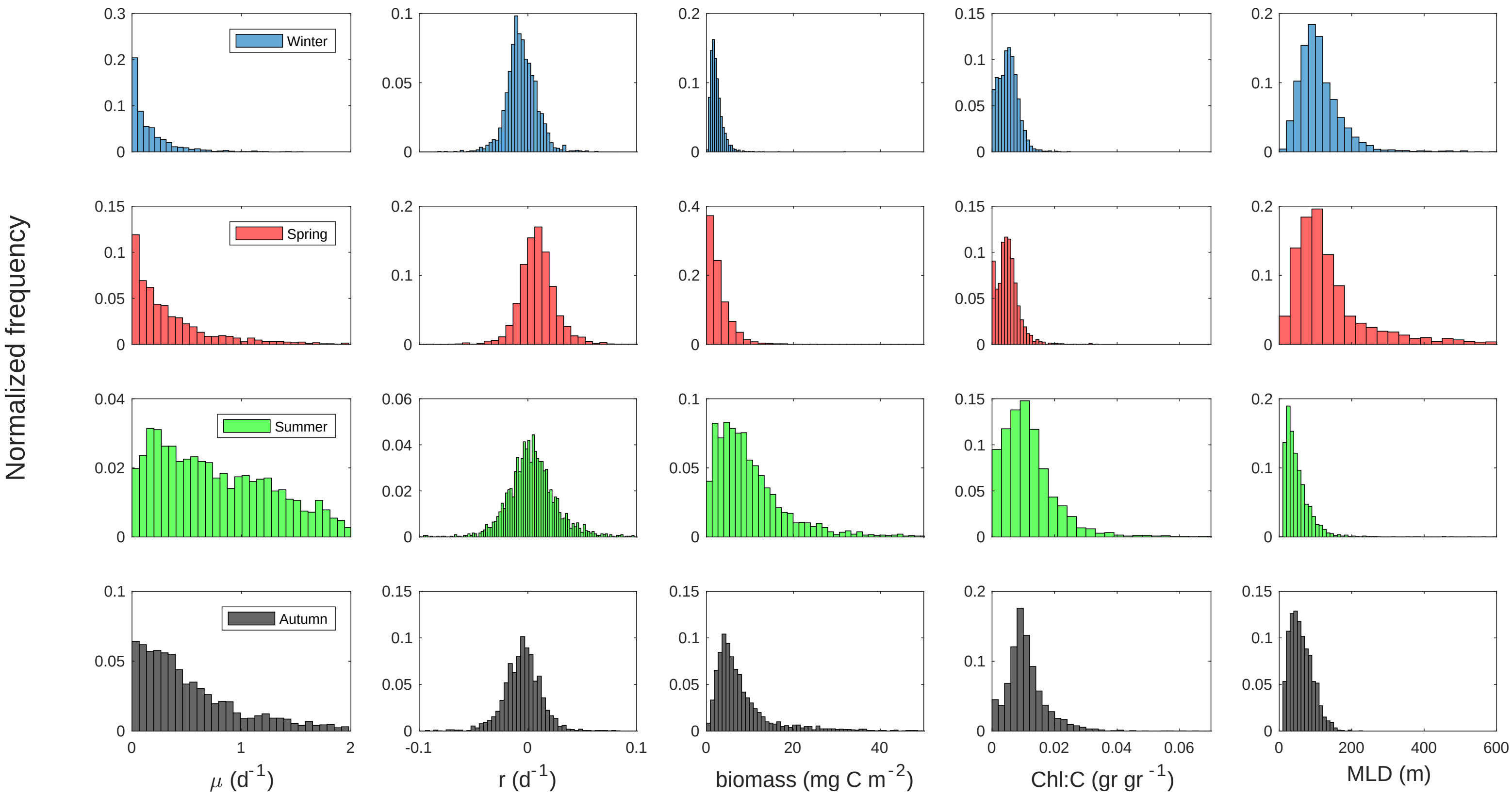

Figure S2: Normalized frequency distribution of float-based phytoplankton variables μ, r, biomass (phytoplankton carbon), Chl:C ratio, and mixed layer depth (MLD), for each season (winter (blue), spring (red), summer (green), and autumn (black)) in the entire Southern Ocean float data set.

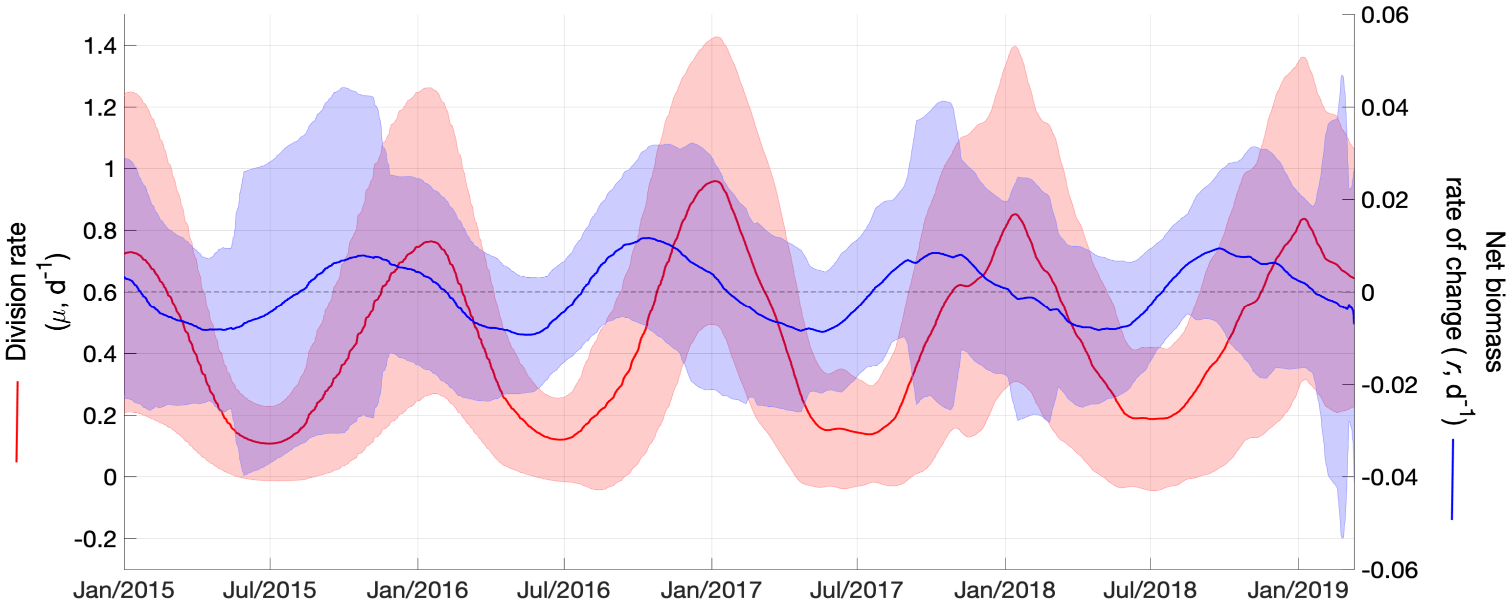

Figure S3: Average time series of modeled phytoplankton division rates (μ) (red continuous line) and phytoplankton net biomass rate of change rate (r) (blue continuous line) with uncertainties represented as the standard deviation of the time series of μ and r (red and blue shaded areas, respectively) (Same as Figure 1c with displayed uncertainties).

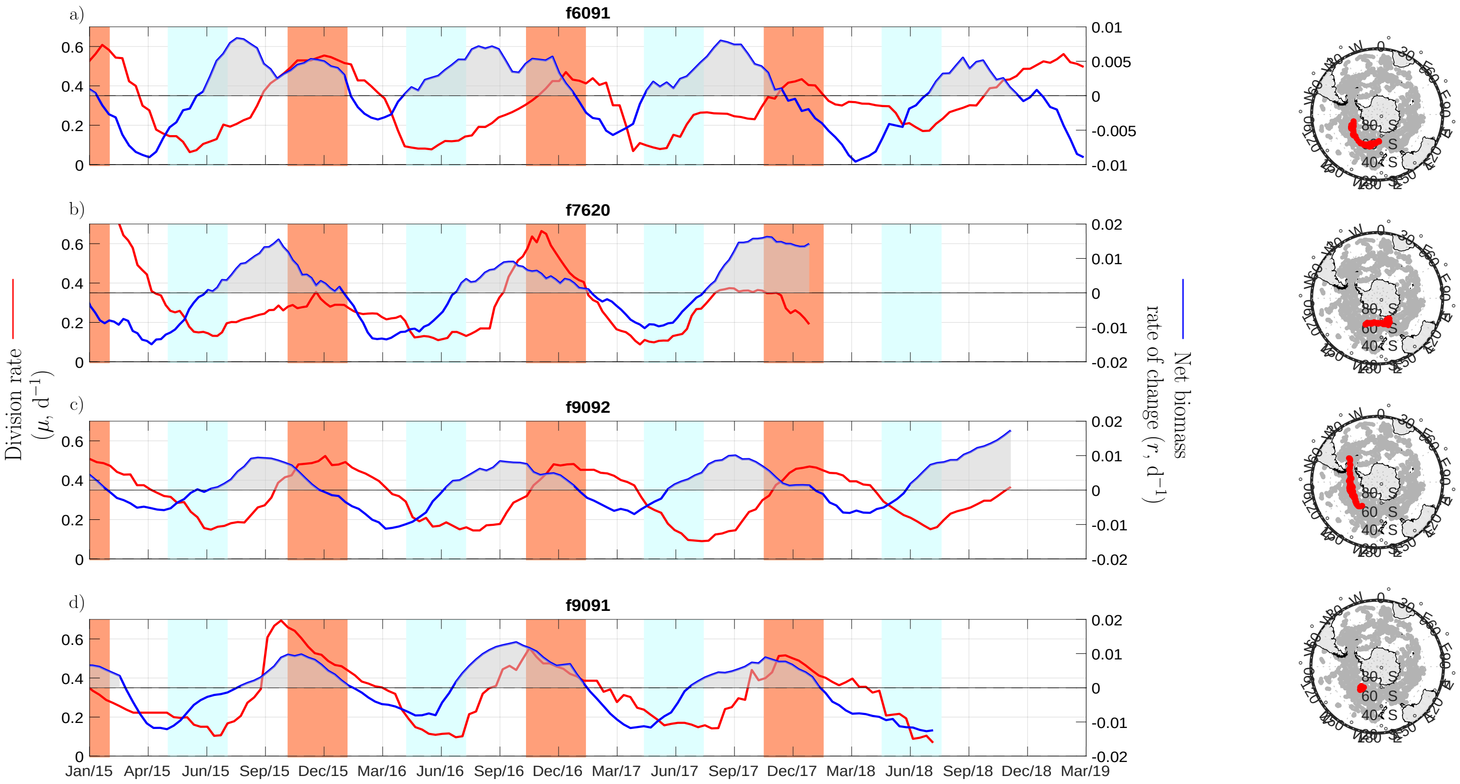

Figure S4: Time series of modeled phytoplankton division rates (μ) and observationally-based net phytoplankton biomass rate of change (r) for individual SOCCOM floats (a) f6091, (b) f7620, (c) f9092, and (d) f9091. Grey shaded areas indicate blooming periods ($r > 0$). Time series have been smoothed using a moving average filter over a 20 days window. Light blue and red shaded panels indicate austral winter (May-August) and summer (November-February) months, respectively. Maps show in red the displacement of each float in the Southern Ocean (trajectories in gray are for all float profiles available in the present SOCCOM data set).

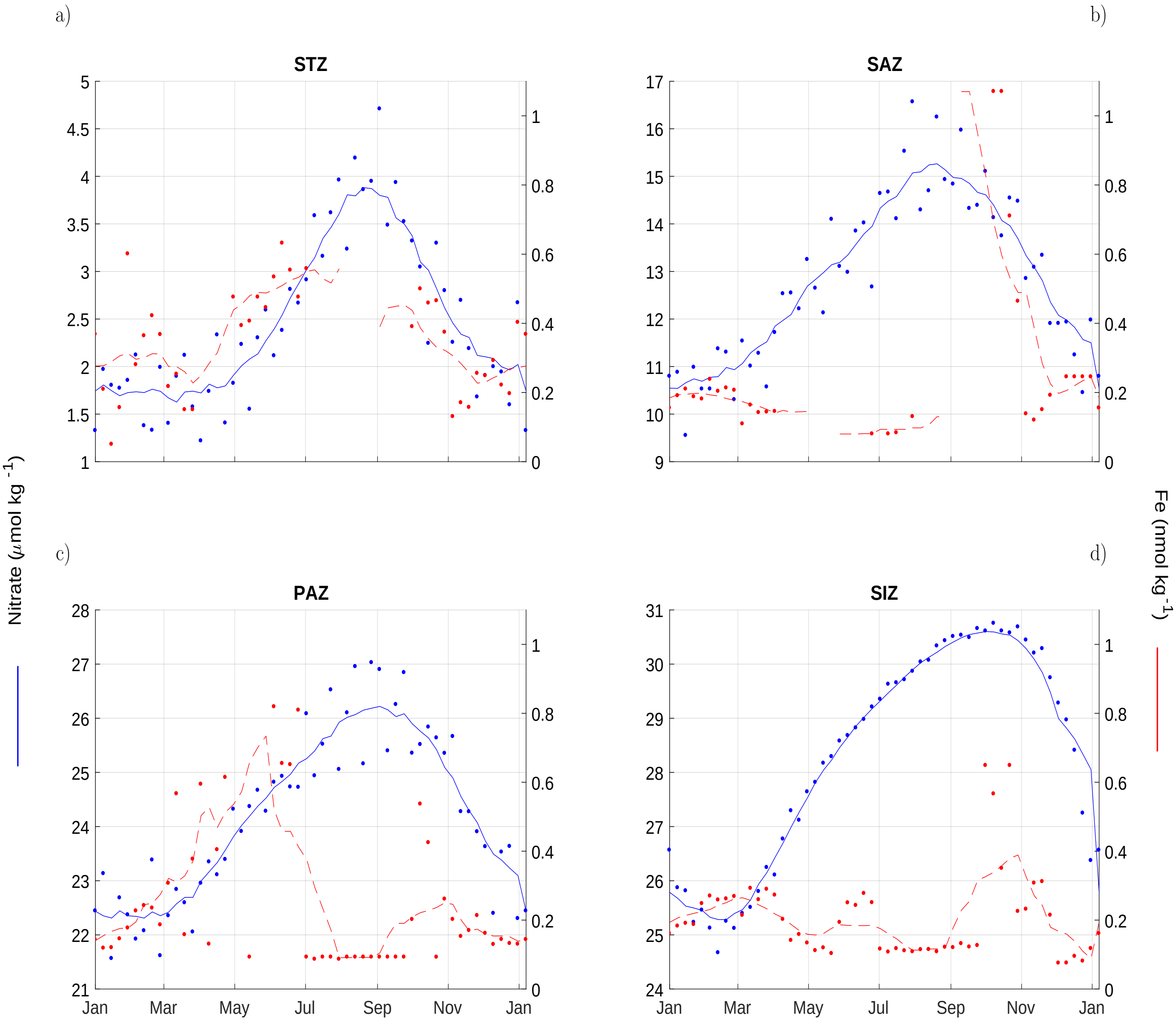

Figure S5: Annual climatology of nitrate and and dissolved iron (Fe) concentration in the surface mixed layer for each environmental zone defined in the Southern Ocean: (a) STZ, (b) SAZ, (c) PAZ, and (d) SIZ. Individual points are weekly averaged observations and continuous line is the result of a smoothing temporal filter over a 60 days window.

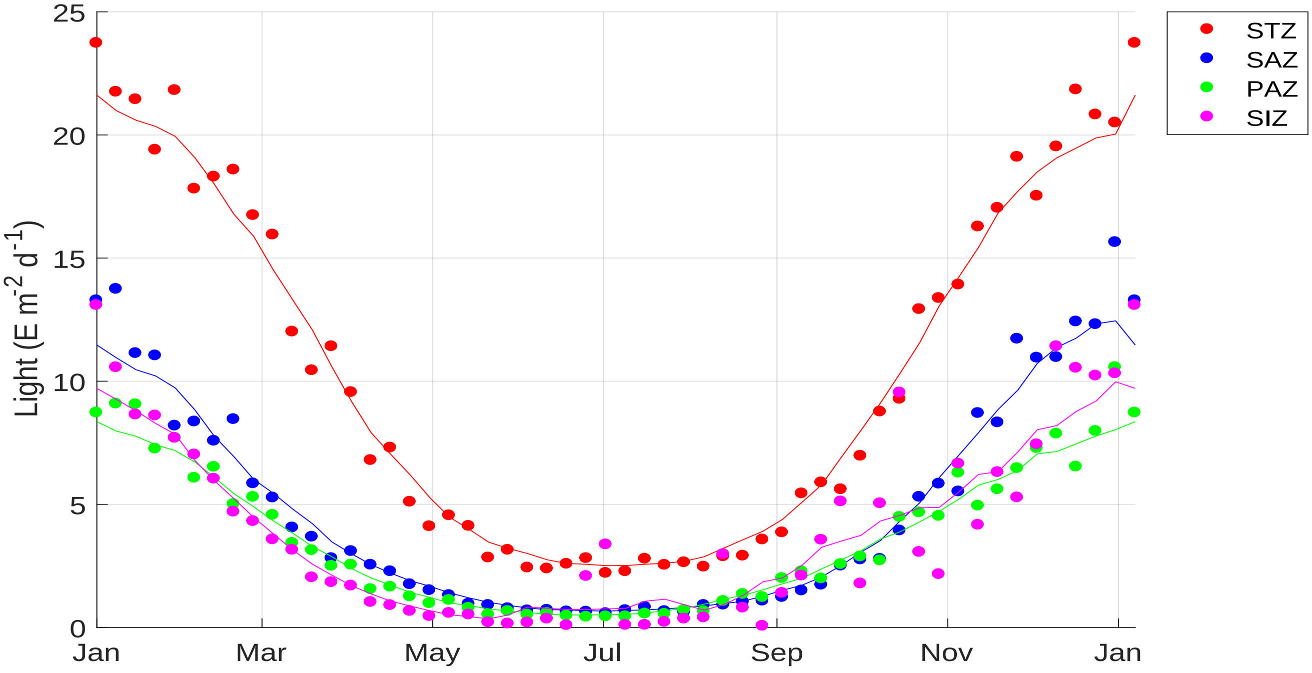

Figure S6: Annual climatology of mean mixed layer light for each environmental zone defined in the Southern Ocean. Individual points are weekly averaged observations and continuous line is the result of a smoothing temporal filter over a 60 days window.

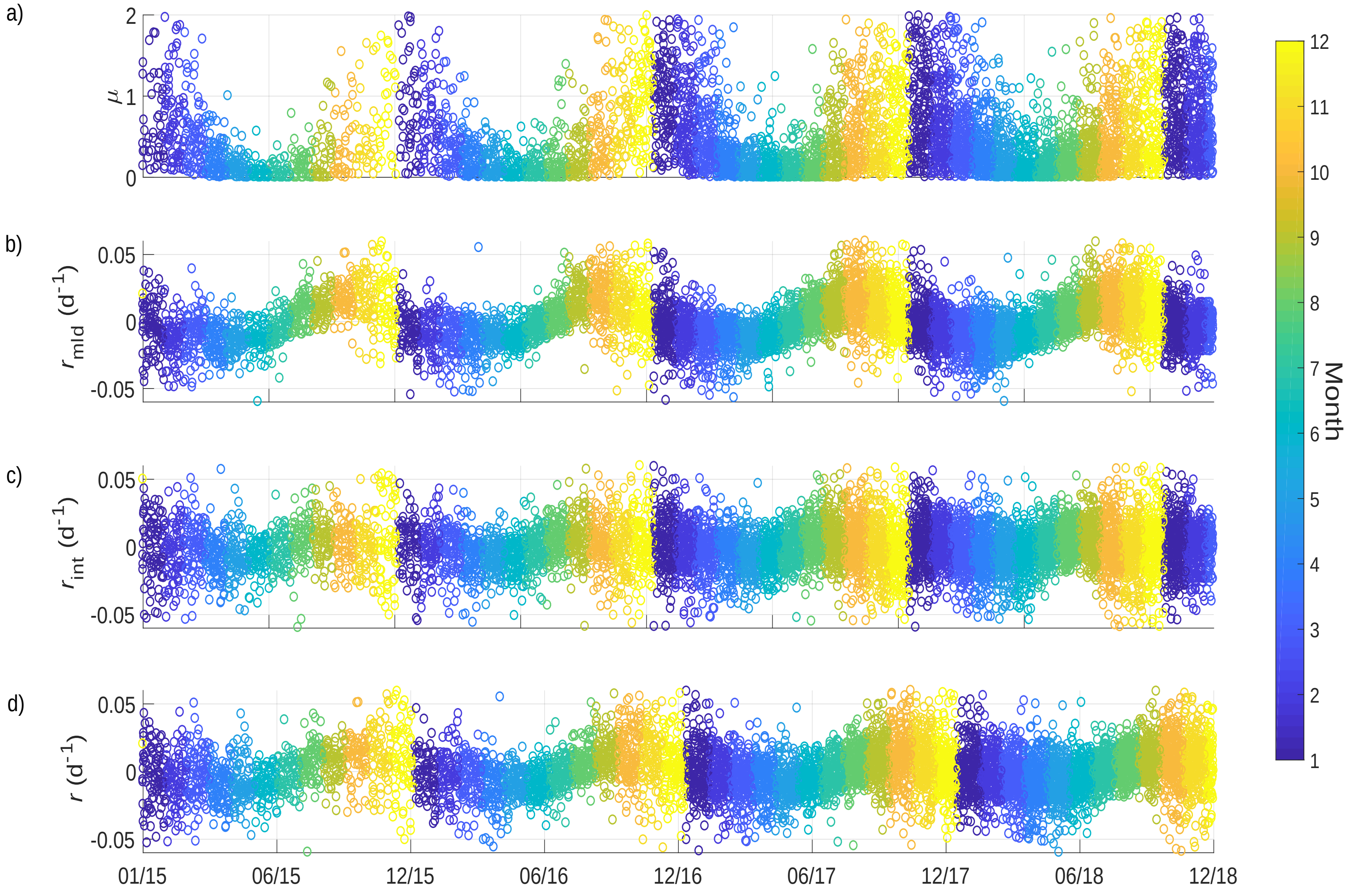

Figure S7: (a) Individual float-based estimates of phytoplankton division rate (μ) from the growth of model employed in this study[9], averaged in the mixed layer. (b) Individual float-based estimates of r based on changes in mixed layer phytoplankton biomass concentration (r_{mld}) and (c) based on the integrated biomass inventory (r_{int}). (d) Final estimate of net accumulation rate (r) based on the switching algorithm (Equation 4). Colorbar indicates the month of each observationally-based estimate.

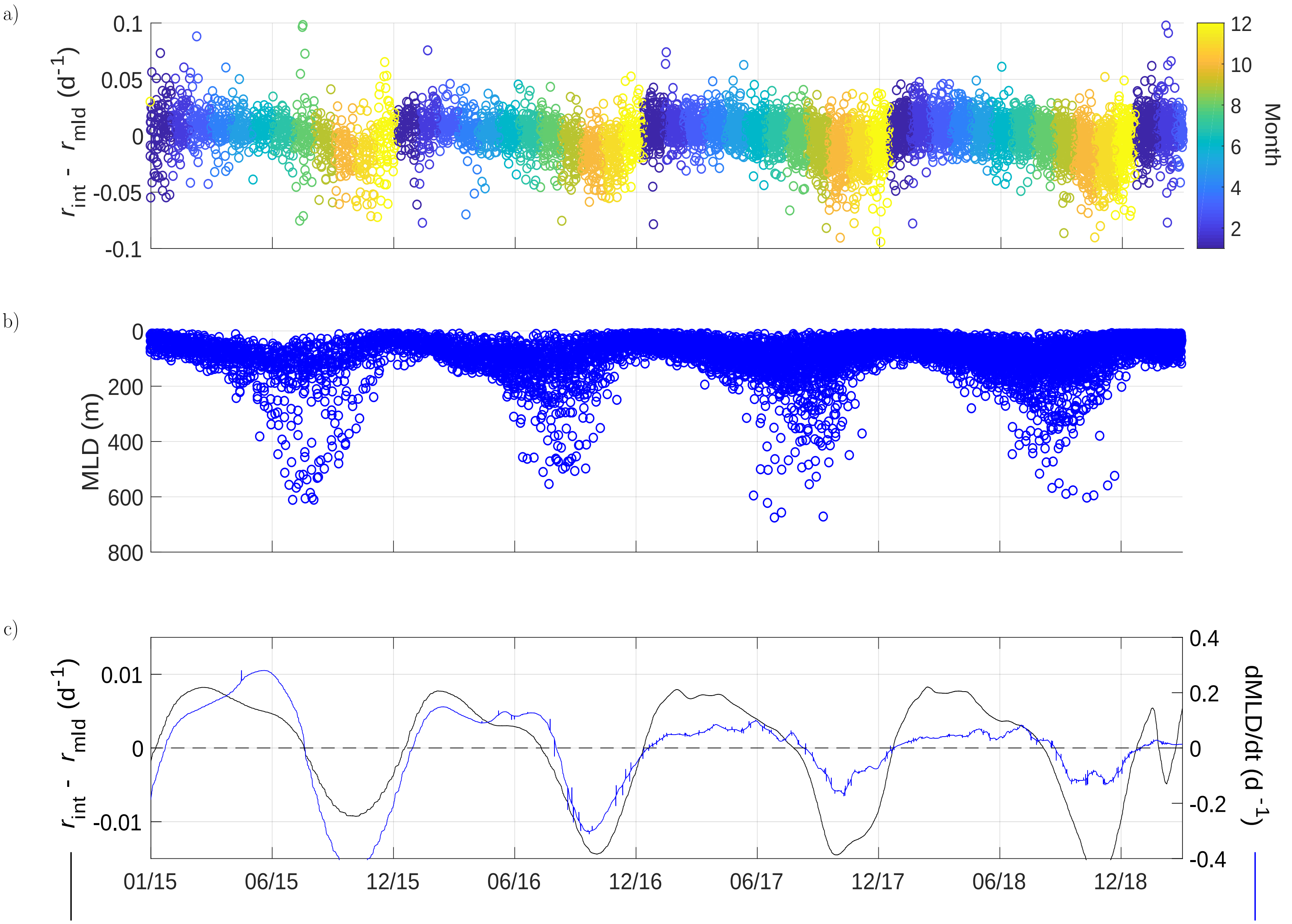

Figure S8: (a) Difference between mean and integrated estimates of the net accumulation rate of phytoplankton biomass (r_{int} - r_{mld}). (b) Individual float-based estimates of mixed layer depth (MLD) based on in situ temperature and salinity profiles[10]. (c) Comparison between smoothed time series of r_{int} - r_{mld} (black continuous line) and the temporal derivative of the mixed layer depth (dMLD/dt) (blue continuous line).

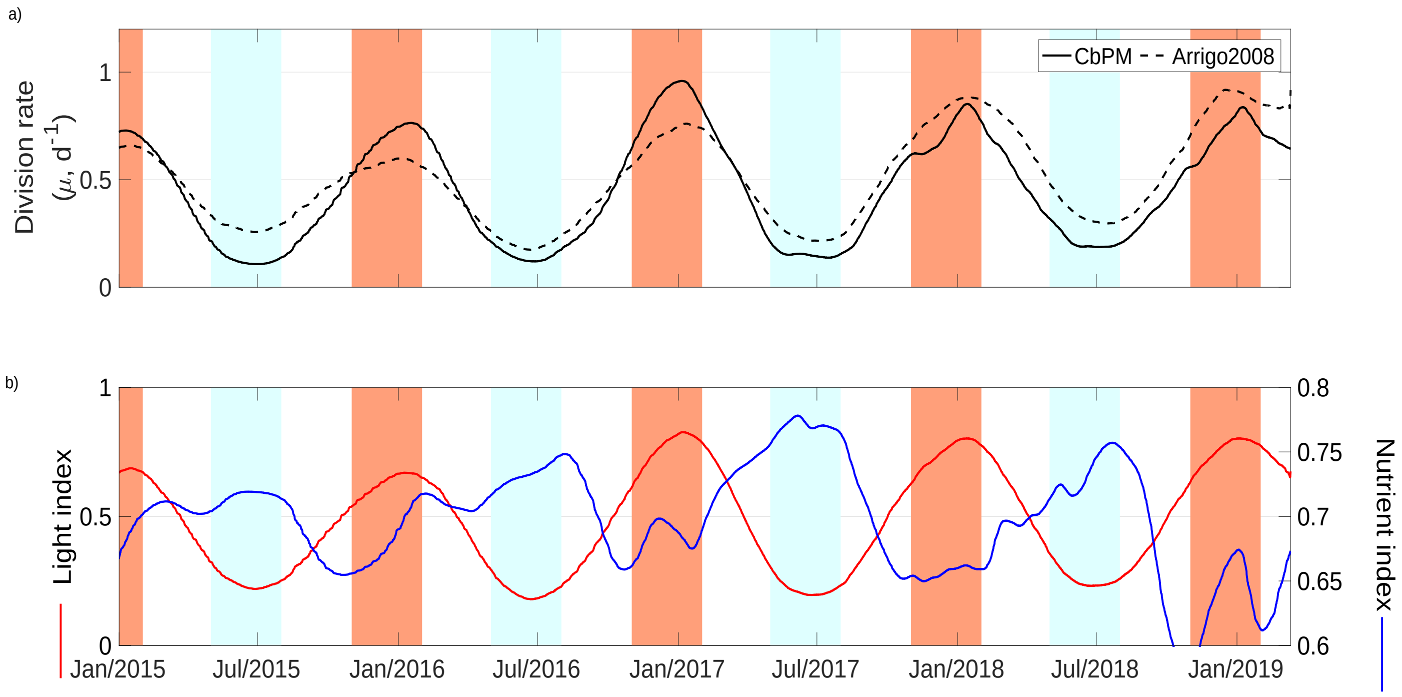

Figure S9: (a) Annual cycles of float-based mean phytoplankton division rates in the upper mixed layer computed from the CbPM (black-continuous line) and the Arrigo2008[4] algorithm (black-dashed line). (a) Annual cycles of the mean mixed layer light (red-continuous line) and nutrient (blue-continuous line) saturation indices of phytoplankton growth inferred from the CbPM.

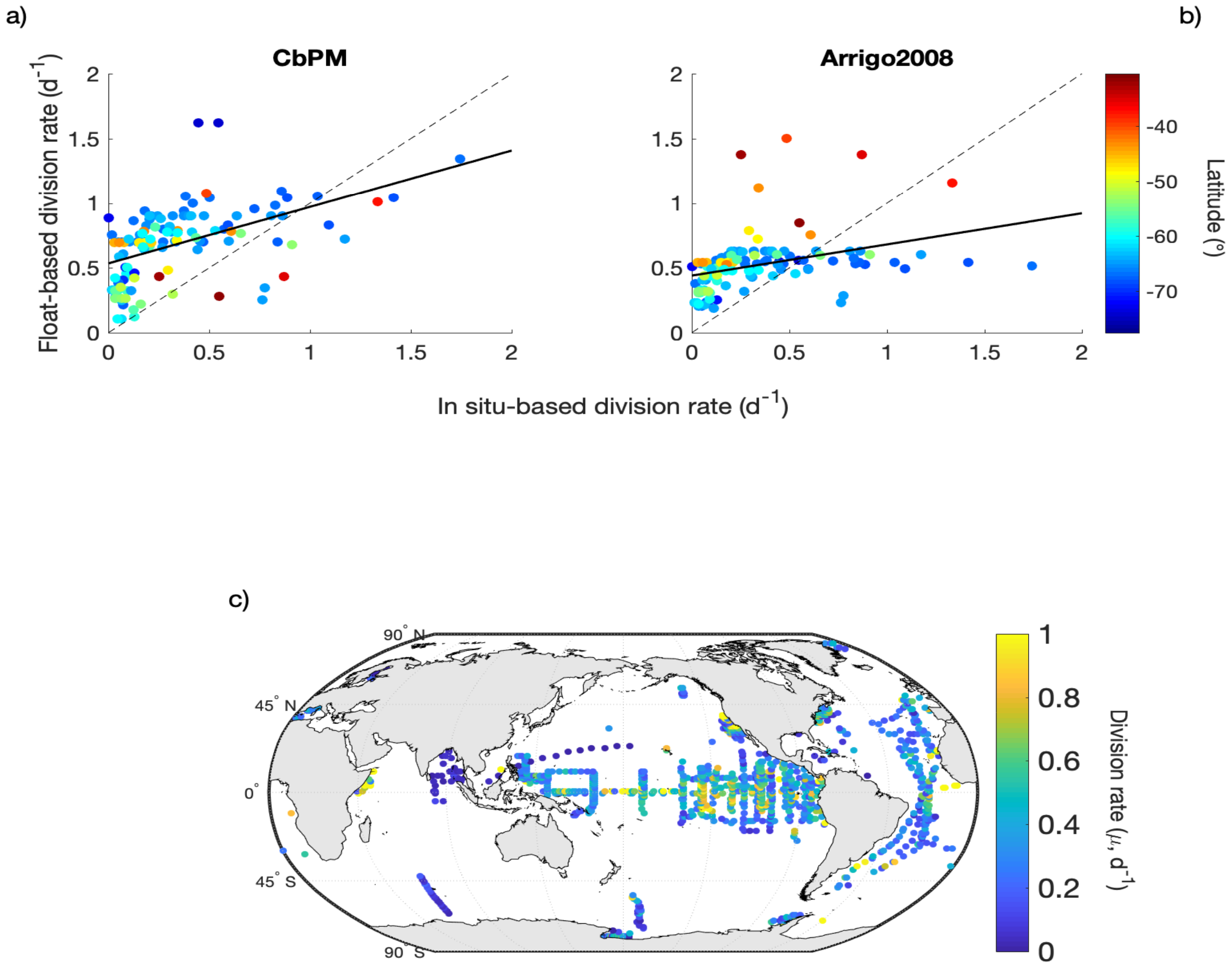

Figure S10: (a) Scatter plot of in situ-based and float-based estimates of upper ocean phytoplankton division rates inferred from the (a) CbPM (R^2 = 0.25, $RMSE_{fit}$ = 0.24 d^{-1}, $RMSE_{model}$ = 0.35 d^{-1}) and (b) the Arrigo2008[4] algorithm (R^2 = 0.13, $RMSE_{fit}$ = 0.19 d^{-1}, $RMSE_{model}$ = 0.58 d^{-1}). Solid black-continuous line is the output from the linear regression model. Black dashed-line represents the one-to-one line. (c) Global patterns of phytoplankton division rates estimated from in situ carbon-14 (^{14}C) based net primary productivity measurements[6] used to validate float based estimates of μ. Modeled outputs of μ in the Southern Ocean were subsampled by averaging all float-based estimates within a horizontal radius of 500 km around each in situ observation at the same month of the year. This resulted in a total of n=187 comparable data points south of 30°S shown in the scatter plots (a and b).

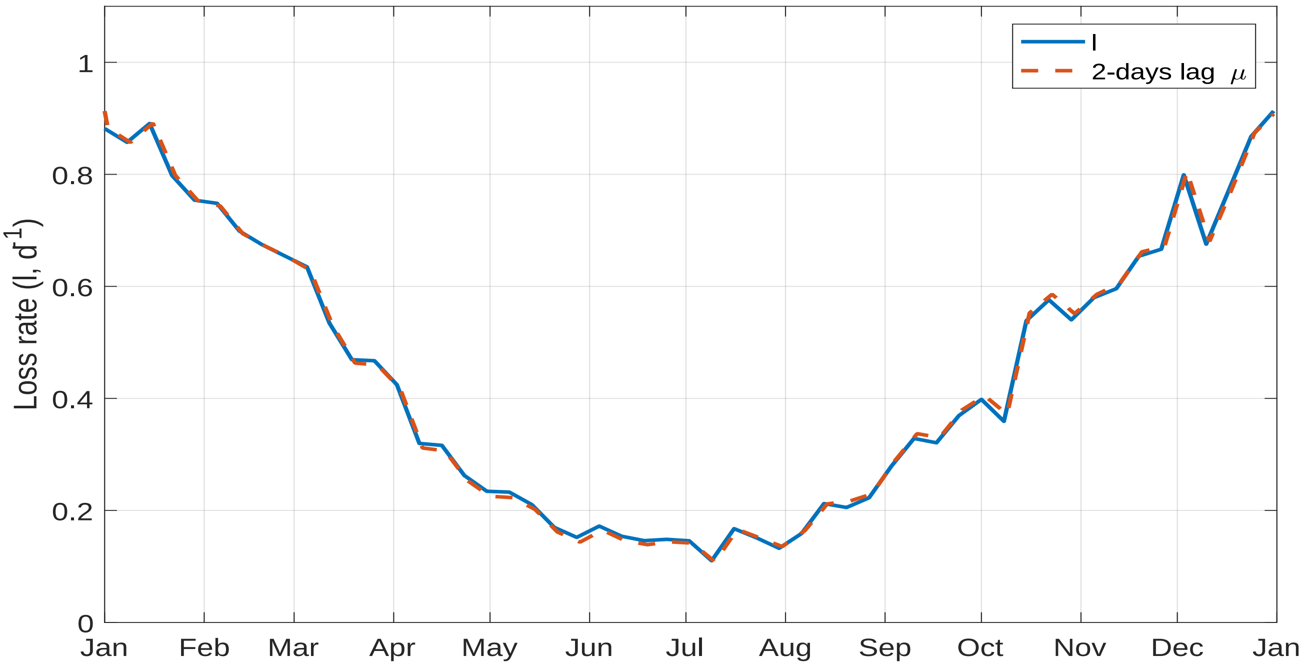

Figure S11: Climatological loss rate (l, blue line) for the Southern Ocean computed from the float-based accumulation and division rate as $l = \mu$ - r. Red dashed-line shows a reconstruction of l as 2-days temporally lagged μ.

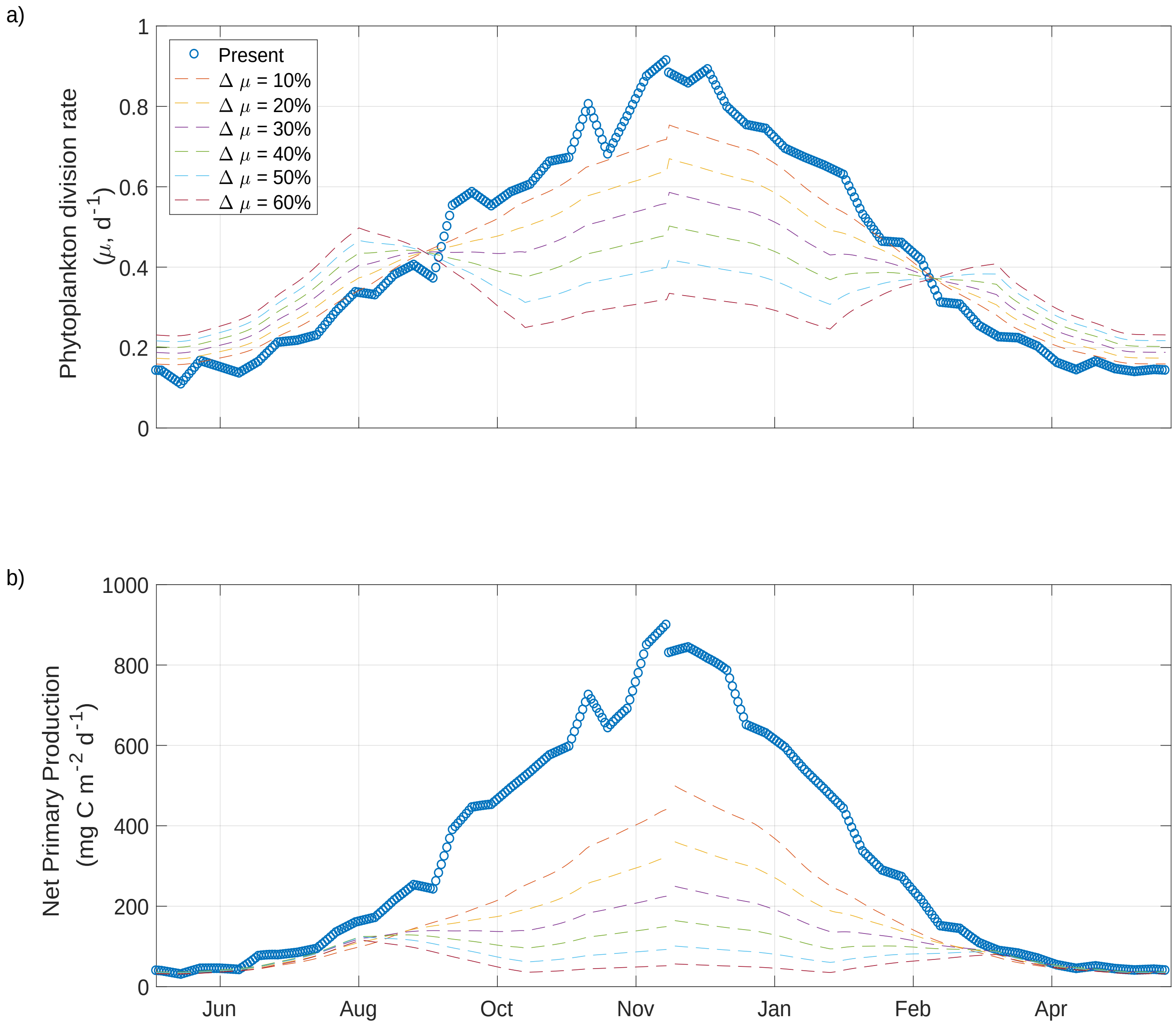

Figure S12: (a) Induced changes in the annual cycle of phytoplankton division rates (μ). (b) Variations in the annual cycle of vertically integrated net primary production (NPP) in the Southern Ocean resulting from relative changes in μ.

References

1. Tagliabue, A. *et al.* A global compilation of dissolved iron measurements: focus on distributions and processes in the Southern Ocean. *Biogeosciences* **9**, 2333–2349 (2012). URL https://www.biogeosciences.net/9/2333/2012/.

2. Boss, E. & Behrenfeld, M. In situ evaluation of the initiation of the North Atlantic phytoplankton bloom. *Geophysical Research Letters* **37** (2010). URL http://dx.doi.org/10.1029/2010GL044174. L18603.

3. Behrenfeld, M. J., Doney, S. C., Lima, I., Boss, E. S. & Siegel, D. A. Reply to a comment by Stephen M. Chiswell on: Annual cycles of ecological disturbance and recovery underlying the subarctic Atlantic spring plankton bloom by M. J. Behrenfeld et al. (2013). *Global Biogeochemical Cycles* **27**, 1294–1296 (2013). URL https://agupubs.onlinelibrary.wiley.com/doi/abs/10.1002/2013GB004720. https://agupubs.onlinelibrary.wiley.com/doi/pdf/10.1002/2013GB004720.

4. Arrigo, K. R., van Dijken, G. L. & Bushinsky, S. Primary production in the Southern Ocean, 19972006. *Journal of Geophysical Research: Oceans* **113** (2008). URL http://dx.doi.org/10.1029/2007JC004551. C08004.

5. Tagliabue, A. *et al.* Surface-water iron supplies in the Southern Ocean sustained by deep winter mixing. *Nature Geoscience* **7**, 314–320 (2014). URL https://doi.org/10.1038/ngeo2101.

6. Buitenhuis, E. T., Hashioka, T. & Qur, C. L. Combined constraints on global ocean primary production using observations and models. *Global Biogeochemical Cycles* **27**, 847–858 (2013). URL http://dx.doi.org/10.1002/gbc.20074.

7. Arteaga, L., Haeëntjens, N., Boss, E., Johnson, K. S. & Sarmiento, J. L. Assess-

ment of export efficiency equations in the Southern Ocean applied to satellite-based net primary production. *Journal of Geophysical Research: Oceans* (2018). URL `https://agupubs.onlinelibrary.wiley.com/doi/abs/10.1002/2018JC013787`. `https://agupubs.onlinelibrary.wiley.com/doi/pdf/10.1002/2018JC013787`.

8. Behrenfeld, M. J. & Boss, E. S. Student's tutorial on bloom hypotheses in the context of phytoplankton annual cycles. *Global Change Biology* **24**, 55–77 (2018). URL `https://onlinelibrary.wiley.com/doi/abs/10.1111/gcb.13858`. `https://onlinelibrary.wiley.com/doi/pdf/10.1111/gcb.13858`.

9. Westberry, T., Behrenfeld, M. J., Siegel, D. A. & Boss, E. Carbon-based primary productivity modeling with vertically resolved photoacclimation. *Global Biogeochemical Cycles* **22**, GB2024 (2008).

10. de Boyer Montégut, C., Madec, G., Fischer, A. S., Lazar, A. & Iudicone, D. Mixed layer depth over the global ocean: An examination of profile data and a profile-based climatology. *Journal of Geophysical Research: Oceans* **109** (2004). URL `http://dx.doi.org/10.1029/2004JC002378`. C12003.
