# Supplementary material for: Seasonal modulation of phytoplankton biomass in the Southern Ocean
Source: Nat Commun. 2020 Oct 23;11:5364. doi: 10.1038/s41467-020-19157-2 (PMC7584623; doi:10.1038/s41467-020-19157-2)
Supplement: Supplementary file 3 — Reporting Summary [file 41467_2020_19157_MOESM3_ESM.pdf]

nature research

Corresponding author(s): Lionel A. Arteaga

Last updated by author(s): Sep 17, 2020

Reporting Summary

Nature Research wishes to improve the reproducibility of the work that we publish. This form provides structure for consistency and transparency in reporting. For further information on Nature Research policies, see Authors & Referees and the Editorial Policy Checklist .

Statistics

For all statistical analyses, confirm that the following items are present in the figure legend, table legend, main text, or Methods section.

n/a	Confirmed	
☐	☒	The exact sample size (n) for each experimental group/condition, given as a discrete number and unit of measurement
☒	☐	A statement on whether measurements were taken from distinct samples or whether the same sample was measured repeatedly
☐	☒	The statistical test(s) used AND whether they are one- or two-sided<br>*Only common tests should be described solely by name; describe more complex techniques in the Methods section.*
☒	☐	A description of all covariates tested
☒	☐	A description of any assumptions or corrections, such as tests of normality and adjustment for multiple comparisons
☐	☒	A full description of the statistical parameters including central tendency (e.g. means) or other basic estimates (e.g. regression coefficient) AND variation (e.g. standard deviation) or associated estimates of uncertainty (e.g. confidence intervals)
☐	☒	For null hypothesis testing, the test statistic (e.g. F, t, r) with confidence intervals, effect sizes, degrees of freedom and P value noted<br>*Give P values as exact values whenever suitable.*
☒	☐	For Bayesian analysis, information on the choice of priors and Markov chain Monte Carlo settings
☒	☐	For hierarchical and complex designs, identification of the appropriate level for tests and full reporting of outcomes
☒	☐	Estimates of effect sizes (e.g. Cohen's d, Pearson's r), indicating how they were calculated

Our web collection on statistics for biologists contains articles on many of the points above.

Software and code

Policy information about availability of computer code

Data collection	All data was collected and analyzed using MATLAB Version: 9.2.0.538062 (R2017a)
Data analysis	All data was collected and analyzed using MATLAB Version: 9.2.0.538062 (R2017a)

For manuscripts utilizing custom algorithms or software that are central to the research but not yet described in published literature, software must be made available to editors/reviewers. We strongly encourage code deposition in a community repository (e.g. GitHub). See the Nature Research guidelines for submitting code & software for further information.

Data

Policy information about availability of data

All manuscripts must include a data availability statement. This statement should provide the following information, where applicable:

- Accession codes, unique identifiers, or web links for publicly available datasets
- A list of figures that have associated raw data
- A description of any restrictions on data availability

BGC-Argo float data are available at the SOCCOM program data portal (http://soccompu.princeton.edu/content/data-access). The specific data set used in this work corresponds to the March 12th 2019 low resolution data snapshot published as a MATLAB data file. Daily global maps of cloud-corrected surface ocean MODIS-Aqua PAR (L3, 4km) are available at the NASA Ocean Color website (https://oceancolor.gsfc.nasa.gov). Dissolved iron data are available at the GEOTRACES International Data Assembly Centre (https://www.bodc.ac.uk/geotraces/data/).

Field-specific reporting

Please select the one below that is the best fit for your research. If you are not sure, read the appropriate sections before making your selection.

Ecological, evolutionary & environmental sciences study design

All studies must disclose on these points even when the disclosure is negative.

Study description	Analysis of seasonal modulations of phytoplankton biomass (large-scale blooms) in the Southern Ocean using observations from biogeochemical-argo floats and satellites.
Research sample	Bio-Argo data from https://soccom.princeton.edu, satellite ocean color from (https://oceancolor.gsfc.nasa.gov). Dissolved iron data from the GEOTRACERS project (https://www.bodc.ac.uk/geotraces/data/).
Sampling strategy	Biogeochemical profiles are obtained autonomously by the bio-argo floats and stored in the SOCCOM data centers, available at https://soccom.princeton.edu. The number of data profiles was determined by the number of biogeochemical profiles available in the March 12th 2019 low resolution data snapshot (https://doi.org/10.6075/J0NV9GM7). The total number of profiles analyzed is 11283, widely distributed across the Southern Ocean (South of 30∘S), which allows for a full characterization of seasonal bloom dynamics in this region.
Data collection	Bio-Argo floats are being constantly deployed through different research cruises in the Southern Ocean. Once deployed, the floats park at 1000 m between profiles, following Argo protocol, and then descend to a maximum depth between 1400 and 2000 m before returning the surface. Profile measurements are made on this ascent and transmitted via the Iridium satellite network at the ocean surface before the float descends back to its park depth. Data is received, stored, and available at https://soccom.princeton.edu.
Timing and spatial scale	Float data corresponds to the period from 06/Mar/2012 to 12/Mar/2019. Profiles are obtained continuously by each float every 5 or 10 days.
Data exclusions	Negative phytoplankton carbon and chlorophyll were excluded from the study in order to avoid spurious outputs from the phytoplankton growth model. Negative phytoplankton carbon represented < 0.001 % of sampled in the upper 200m. Negative chlorophyll estimates represented < 0.01 % of the entire float data set.
Reproducibility	Physical experiments were not conducted in this study. Numerical and data analyses were replicated multiple times to ensure consistency and remove computational coding errors.
Randomization	All available data was used, no randomization procedure was necessary in our study.
Blinding	No interventional experiment that required blinding was conducted in this study.

Did the study involve field work? ☐ Yes ☒ No

Reporting for specific materials, systems and methods

We require information from authors about some types of materials, experimental systems and methods used in many studies. Here, indicate whether each material, system or method listed is relevant to your study. If you are not sure if a list item applies to your research, read the appropriate section before selecting a response.

Materials & experimental systems

n/a	Involved in the study
☒	☐ Antibodies
☒	☐ Eukaryotic cell lines
☒	☐ Palaeontology
☒	☐ Animals and other organisms
☒	☐ Human research participants
☒	☐ Clinical data

Methods

n/a	Involved in the study
☒	☐ ChIP-seq
☒	☐ Flow cytometry
☒	☐ MRI-based neuroimaging
